# A core curriculum of point-of-care ultrasound examinations for frontline physicians in primary care: results from a European Delphi study

**DOI:** 10.1055/a-2590-5242

**Published:** 2025-06-04

**Authors:** Camilla Aakjær Andersen, Thomas Løkkegaard, Leizl Joy Nayahangan, Hazel Edwards, Mihai Sorin Iacob, Kristina Lebedevaite, Mateusz Kosiak, Elena Codruta Gheorghe, Adib Salim, Viktor Rüttermann, Caroline Ewertsen, Christian Jenssen

**Affiliations:** 1 1004 Center for General Practice, Aalborg University, Aalborg, Denmark; 2 4321 CAMES Rigshospitalet, University of Copenhagen, Copenhagen, Denmark; 3 459239 BMUS, British Medical Ultrasound Society, London, United Kingdom of Great Britain and Northern Ireland; 4 162271 Centre for Preventive Medicine, University of Medicine and Pharmacy Victor Babes Timisoara, Timisoara, Romania; 5 Antalgija, Medical Practice, Kaunas, Lithuania; 6 Sonographic educational platform, Eduson, Gdansk, Poland; 7 121532 University of Medicine and Pharmacy of Craiova, Craiova, Romania; 8 9333 Pediatrics, ASST Papa Giovanni XXIII, Bergamo, Italy; 9 Hausärztliche Gemeinschaftspraxis Nordholter Weg, Medical Practice, Drensteinfurt, Germany; 10 Department of Radiology, Copenhagen University Hospital, Rigshospitalet, Copenhagen OE, Denmark; 11 Brandenburg Institute of Clinical Ultrasound (BICUS), Medical University Brandenburg, Neuruppin, Germany

**Keywords:** Point-of-care Ultrasound, General Practice, Family Medicine, Primary Healthcare, Curriculum

## Abstract

Point-of-care ultrasound (PoCUS) has found its way into primary care in some, but not all, European countries. A prerequisite for achieving Europe-wide comparable diagnostic reliability of PoCUS performed by primary care physicians is high-quality training that is limited to relevant, frequently encountered PoCUS applications that are easy to learn and master. A European Federation of Societies for Ultrasound in Medicine (EFSUMB) task force performed a brainstorming exercise to identify all possible ultrasound examinations that could be performed in primary care. A 3-stage Delphi process was launched. The Delphi panelists were 95 primary care physicians from 28 European countries with more than 2 years of experience using and teaching ultrasound. Solely focusing on the complexity of performing PoCUS, the panelists reduced the brainstorming list in a stepwise manner to a basic core curriculum intended for primary care frontline physicians including 40 diagnostic PoCUS examinations within 13 different anatomical areas and no ultrasound-guided procedures. A 75% cut-off was used for agreement. Despite the great heterogeneity of the Delphi panel representing different views and contexts from across Europe, kappa statistics showed substantial or moderate agreement across Delphi rounds 2 and 3 for 85% of the 40 diagnostic PoCUS applications. The results of this study offer guidance for EFSUMB to establish training recommendations for a basic core curriculum that can be adapted to the needs of different regions of Europe and thus create a basis for PoCUS to become a reliable diagnostic tool in primary care across Europe, based on common quality standards.

## Zusammenfassung

Point-of-Care-Ultraschall (PoCUS) hat in einigen, aber nicht allen europäischen Ländern Einzug in die Primärversorgung gehalten. Voraussetzung für eine europaweit vergleichbare diagnostische Zuverlässigkeit des PoCUS, wenn er von Ärzten in der Primärversorgung durchgeführt wird, ist eine hochwertige Ausbildung, die sich auf relevante, häufige PoCUS-Anwendungen beschränkt, die leicht zu erlernen und zu beherrschen sind. Eine Arbeitsgruppe der European Federation of Societies for Ultrasound in Medicine (EFSUMB) führte ein Brainstorming durch, um alle möglichen Ultraschall-Untersuchungen zu ermitteln, die in der Primärversorgung durchgeführt werden könnten. Es wurde ein 3-stufiges Delphi-Verfahren gestartet. Die Delphi-Panel-Teilnehmer waren 95 Hausärzte aus 28 europäischen Ländern mit mehr als 2 Jahren Erfahrung in der Anwendung und in der Lehrtätigkeit im Bereich des Ultraschalls. Die Panel-Teilnehmer konzentrierten sich ausschließlich auf die Komplexität der Durchführung des PoCUS und reduzierten die Brainstorming-Liste schrittweise auf ein Basis-Curriculum für Ärzte in der Primärversorgung, das 40 diagnostische PoCUS-Untersuchungen in 13 verschiedenen anatomischen Bereichen, ohne ultraschallgeführte Verfahren, umfasst. Als Schwellenwert für die Zustimmung wurde eine Quote von 75% festgelegt. Trotz der großen Heterogenität des Delphi-Gremiums, das unterschiedliche Ansichten und Kontexte aus ganz Europa repräsentierte, zeigte die Kappa-Statistik in den Delphi-Runden II und III für 85% der 40 diagnostischen PoCUS-Anwendungen eine erhebliche oder mittlere Übereinstimmung. Die Ergebnisse dieser Studie bieten der EFSUMB eine Orientierungshilfe für die Erstellung von Ausbildungsempfehlungen für ein Basis-Curriculum, das an die Bedürfnisse der verschiedenen Regionen Europas angepasst werden kann. Somit wird die Grundlage dafür geschaffen, dass PoCUS auf der Basis gemeinsamer Qualitätsstandards zu einem zuverlässigen Diagnose-Werkzeug in der Primärversorgung in ganz Europa wird.

## Introduction

The use of ultrasound as part of the initial examination and assessment of patients is increasing across medical specialties. This is due to the greater availability of compact, low-cost, high-quality ultrasound scanners 1 2 3 , the introduction of point-of-care ultrasound (PoCUS) as a focused examination designed for clinicians 4 5 6 , and the introduction of ultrasound in the curriculum of medical schools and in resident training 7 .

Ultrasound examinations have also been introduced in primary care 8 . Frontline physicians such as general practitioners, family medicine doctors, pediatricians, and palliative care physicians are increasingly using the technology. This proliferation in use also drives the need for education and decision tools to help frontline physicians working in primary care in terms of what to scan and, equally important, what not to scan 9 . This is in accordance with the concept of professionalism of the European Federation of Societies for Ultrasound in Medicine and Biology (EFSUMB), which aims to make medical ultrasound a reliable professional service based on common and uniform quality standards – regardless of where it is performed and who performs the scan and other variable conditions in each national healthcare system 10 .

There is considerable variation in the organization of primary healthcare between countries 11 , in the access to ultrasound equipment in primary care 12 , and in the training and specialization of primary care doctors 13 14 15 . An extensive curriculum has been proposed for family medicine residents in the United States of America 16 , but guidance is still missing for office-based frontline physicians working in primary care. Developing an ultrasound curriculum for primary care based on contextual factors like relevance of an examination in the diagnostic process, feasibility of performing the examination in the primary care setting, or frequency of patient encounters will be difficult as primary care physicians (PCPs) are a heterogeneous group, and the organization of primary care differs. In contrast, the complexity of performing a given ultrasound examination and the competence level required to do so are more generic and generalizable.

In some European countries, PoCUS is already well integrated into the training and clinical practice of frontline physicians working in primary care. In other countries, there are significant barriers to implementation 17 . A uniform core curriculum may support efforts to establish PoCUS training in countries and develop the basis for a common standard in this important part of clinical diagnostic ultrasound.

Frontline physicians working in European primary care, who use and teach ultrasound will have accumulated valuable experience as to which ultrasound examinations are easy to learn and master, and which ultrasound scans require more training and experience. In this study, we aim to use this knowledge to develop a prioritized list of ultrasound examinations that could be included in a basic ultrasound curriculum for frontline physicians working in primary care based on the Delphi technique.

## Methods

### Study design

We conducted a systematic general needs assessment to explore agreement on which ultrasound examinations could be included in a basic ultrasound curriculum for frontline physicians working in primary care family medicine 18 . A Delphi process was used to achieve consensus among a group of primary care physicians, who are experienced ultrasound users and experienced ultrasound teachers, regarding which examinations to include in a basic ultrasound curriculum 19 .

### Delphi panel participants

#### Recruitment of Delphi panel members

We aimed to have a large heterogeneous panel of experts for our study including between 1–10 Delphi panel members from each European country with more than 100,000 inhabitants (excluding Andorra, Monaco, Liechtenstein, San Marino, The Vatican City, The Faroe Islands, and Greenland).

Between May 2023 and October 2023, an invitation letter describing the study was sent to the national ultrasound societies registered in EFSUMB, through family medicine networks (World Organization of Family Doctors [WONCA], European Network on Prevention and Health Promotion in Family Medicine and General Practice [EUROPREV], European General Practice Research Network [EGPRN]), and the European Association for the Development of Clinical Ultrasonography in Ambulatory Health Care (EUVEKUS/EADUS), was posted on the EFSUMB homepage, and was sent to the corresponding authors of research papers, describing the use of ultrasound in European general practice. In countries with no formal ultrasound societies, the invitation was sent to informal interest groups, course providers, or ultrasound networks. In addition, the authors mentioned the study at scientific conferences and circulated the invitation broadly in their personal networks.

The invitation included a small recruitment questionnaire for potential participants with questions related to the inclusion and exclusion criteria.

#### Selection of Delphi panel members

Delphi panel members were selected based on their responses to the recruitment questionnaire. To be eligible to participate as a Delphi panel member, the European frontline physicians had to have (1) more than 2 years of clinical experience as a frontline physician working in primary care (e.g., general practitioner, family medicine specialist, pediatrician, palliative care doctor), (2) more than 2 years of experience using ultrasound in the primary care setting, (3) experience as an ultrasound tutor or teacher, and (4) the ability to read, understand, and write English, as participants were expected to be able to read and understand the international scientific literature. Participants with potential conflicts of interest (e.g., financial) were excluded. To ensure heterogenicity and avoid overrepresentation from one country, we included a maximum of 10 participants from each country. If there were more than 10 eligible participants from the same country, we randomly selected 10 and excluded the others.

The identity of each participant was kept anonymous from other participating PCPs.

### The Delphi process

A task force was appointed by EFSUMB to develop a core curriculum for European primary care doctors. During a brainstorming phase, the task force developed a comprehensive list of ultrasound scans ( **Supplemental file 1** ), which was used as a foundation for a 3-round Delphi process (Supplemental ).

For this, we defined **a diagnostic ultrasound examination** as a PoCUS examination of a specific organ or structure performed by the frontline physician to specifically rule out or rule in a certain disease entity, e.g., gallstones or residual urine, and **an ultrasound-guided procedure** as the use of ultrasound by the frontline physician to direct a needle to the correct location, e.g., intra-articular injections, paracentesis/thoracocentesis.

#### Delphi round 1 – assessing the level of competence

After inclusion, the selected Delphi panel members received an e-mail with a link to the online questionnaire and instructions on how to complete it. In the questionnaire, participants were asked to provide the following information about themselves: Age (years), gender (F/M/other), country, medical specialization, experience working as a primary care physician (years), experience with ultrasound in the primary care setting (years), and experience as an ultrasound teacher or tutor (years). In addition, participants were asked for the following information about their country: Organization of primary care in the country (public/private/other), ultrasound training currently part of medical school in the country (yes/no/other), ultrasound training currently part of specialist training for primary care physicians in the country (yes/no/other), and an estimate of the number of primary care physicians using ultrasound in the country (%).

Next, panel members were presented with the preliminary list of ultrasound examinations and procedures and asked to determine: “ *Which level of scanning competence is required to perform the following ultrasound examinations* ”. Corresponding to the EFSUMB levels of scanning competence 19 , the panel members were instructed to choose “basic level of scanning competence”, if they felt that a particular ultrasound examination or procedure was easy to learn and master, “advanced level of scanning competence”, if they felt that it required more training and practice, and “expert level of scanning competence”, if they felt that it required extensive training, practice, and experience.

The panel members were asked to focus solely on the complexity of performing the scan and to select ultrasound examinations without regard to resources, relevance, or feasibility in primary care, as we aimed to minimize contextual factors in their assessment.

### Delphi round 2

In the next Delphi round, panel members were introduced to the overall results from the first round. They were asked to assess each included ultrasound examination and determine for each examination whether *‘This ultrasound examination should be included in a basic core curriculum for frontline physicians working in primary care’.* The panel members evaluated this using a 5-point Likert scale where 5 equaled ‘strongly agree’ and 1 equaled ‘strongly disagree’.

### Delphi round 3

In the 3 ^rd^ Delphi round, panel members were introduced to a pre-prioritized list of ultrasound examinations that had been ranked based on the ratings gathered in the previous rounds. Panel members were asked to carefully review the ultrasound examinations and re-rate the examinations by answering the same question as in Delphi round 2.

### Data analysis

All panel members had an equal voice in the analysis. Data were collected using SurveyXact (Rambøll, Aarhus, Denmark) and transferred to STATA (Stata Statistical Software: Release 17. College Station, TX: StataCorp LLC) for statistical analysis.

Participant and country characteristics were collected as categorical and continuous variables and characterized using descriptive statistics. Results from Delphi rounds 1–3 were characterized using absolute numbers and percentages. In addition, the mean Likert score was calculated in rounds 2 and 3. The consensus level was set to 50% in round 1, 65% in round 2, and 75% in round 3. The stability of each respondent’s reply across rounds 2 and 3 was determined by kappa statistics 20 .

### Ethics, personal data, and dissemination

Eligible participants provided informed consent to participate in the study. During data collection and analysis, only CAA had access to the contact information of the participants. All data were anonymized following the data analysis. Data were stored in accordance with the guidelines from the Danish Data Protection Agency. As this study solely collected questionnaire-based data, no approval was needed from the ethics committee according to Danish legislation.

## Results

**Fig. 1:**
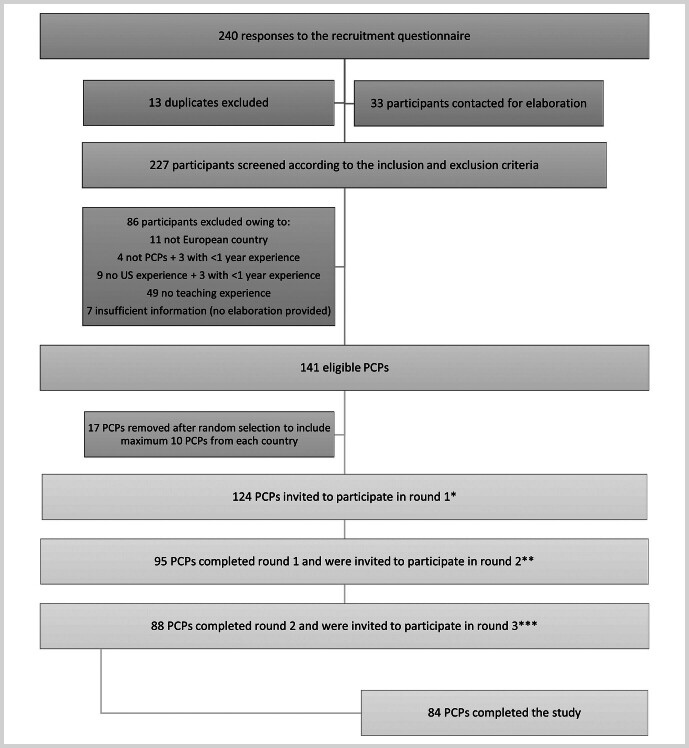
Recruiting the Delphi panel. PCP = primary care physicians, US = ultrasound * Survey round 1 was distributed on Dec 18 ^th^ , 2023. A reminder followed on Jan 3 ^rd^ and 11 ^th^ , 2024. The Survey closed on Jan 22 ^nd^ , 2024. ** Survey round 2 was distributed on Feb 1 ^st^ , 2024. A reminder followed on Feb 10 ^th^ , Feb 26 ^th^ , and Mar 11 ^th^ , 2024. The survey closed on Mar 18 ^th^ , 2024. *** Survey round 3 was distributed on Mar 26 ^th^ , 2024. A reminder followed on Apr 4 ^th^ , 15 ^th^ , 26 ^th^ and May 13 ^th^ , 2024. The survey closed on May 25 ^th^ , 2024.

A total of 240 responded to the survey invitation and recruitment questionnaire. From this, 124 eligible primary care doctors from 28 European countries were invited to participate in the Delphi process. 95 (76.6%) participants from 25 European countries, 88 (70.9%) participants from 24 countries, and 84 (67.7%) participants from 23 countries completed the 1 ^st^ , 2 ^nd^ , and 3 ^rd^ Delphi rounds, respectively ( Fig. 1 ). Background information on participants is provided in Table 1 .

**Table:** **Table 1** Background information on participants.

	N (%)	Min.–max	Mean (SD)
**Age (years)**		26–69	45.1 (10.0)
**Gender (male)**	69 (72.6)		
**Experience as PCP (years)**		2–35	14.6 (9.2)
**Experience using ultrasound (years)**		1–34	10.3 (7.5)
**Experience as ultrasound instructor (years)**		1–35	6.4 (6.0)
Medical specialty*			
Cardiology	1 (0.8)		
Emergency medicine	11 (8.5)		
Endocrinology	2 (1.5)		
Family medicine/general practitioner	76 (58.5)		
Gastroenterology	9 (6.9)		
Geriatric medicine	3 (2.3)		
Hematology	2 (1.5)		
Internal medicine	9 (6.9)		
Neurology	2 (1.5)		
Obstetrics and gynecology	4 (3.1)		
Oncology	1 (0.8)		
Palliative medicine	3 (2.3)		
Pediatrics	4 (3.1)		
Physical medicine and rehabilitation	1 (0.8)		
Preventive medicine	3 (2.3)		
Psychiatry	1 (0.8)		
Radiology	1 (0.8)		
Respiratory medicine	1 (0.8)		
Rheumatology	1 (0.8)		
Sports medicine	3 (2.3)		
Background information on participants included in Delphi round 1 (N = 95) PCP = Primary care physician * Some participants registered as having more than one medical specialty.			

Table 2 provides the participants’ characterization of current ultrasound dissemination in their country. According to the participants, there was a large variation in the estimated use of ultrasound in primary care from 1% in Slovakia and Croatia to 30–80% in Germany and 10–65% in Spain. In 17 countries, participants stated that ultrasound training was either an optional or mandatory part of medical school depending on regional conditions/regulations. In Slovakia, ultrasound training was a mandatory part of PCP training, in Slovenia, mandatory ultrasound training for PCP trainees is to be introduced in 2025, and in 15 other countries, ultrasound training for PCP trainees was declared to be optional or depends on regional conditions/regulations.

**Table:** **Table 2** Characteristics of participating countries.

Country	Number of participants in each Delphi round (N rounds 1/2/3)	Estimated percentage of PCPs using ultrasound# Min.–max. (mean) %	Ultrasound training part of medical school curriculum# (% yes)	Ultrasound training part of PCP training curriculum# (% yes)	Organization of primary healthcare#
Austria	4/4/3	10–20 (13.8)	100.0*	100.0*	Mixed, private or public
Belgium	3/3/3	8–15 (11.0)	0	30.0*	Mixed or private
Croatia	1/1/0	1	0	0	Mixed or private
Czechia	4/4/4	5–10 (7.5)	25.0*	25.0*	Mixed or private
Denmark	10/9/9	10–20 (15.3)	80.0**	40.0*	Public
Finland	3/3/3	5–30 (16.7)	100.0*	0	Mixed or public
France	10/10/10	1–16 (6.1)	50.0*	80.0*	Mixed, private or public
Germany	10/9/8	30–80 (58.5)	90.0**	90.0**	Mixed, private or public
Greece	1/1/1	10	0	0	Mixed
Hungary	1/0/0	5	0	0	Public
Iceland	2/2/2	30 (30.0)	50.0*	50.0*	Mixed or public
Ireland	1/1/1	2	0	0	Mixed
Italy	9/8/7	1–40 (16.6)	22.2*	33.3**	Public
Moldova	1/1/1	10	100.0*	100.0*	Mixed
Netherlands	2/2/2	5 (5.0)	0	0	Public
Norway	5/4/4	4–40 (18.5)	40.0**	100.0*	Mixed or public
Poland	4/4/4	10–25 (15.0)	25.0*	75.0**	Mixed or public
Romania	5/4/4	20–35 (26.8)	80.0**	80.0**	Mixed, private or public
Slovakia	1/1/1	1	0	100.0	Public
Slovenia	1/1/1	10	100.0*	100.0***	Public
Spain	7/7/7	10–65 (30.0)	42.9*	85.7**	Mixed or public
Switzerland	3/3/3	30–40 (36.7)	100.0**	100.0**	Mixed or private
Turkey	1/1/1	20	100.0*	100.0*	Mixed
Ukraine	1/1/1	5	0	0	Mixed
United Kingdom	5/4/4	0.1–5 (1.5)	20.0*	0	Mixed or Public
PCP = primary care physicians ^#^ Declared by the participating PCPs in this study * Ultrasound training is optional or depends on regional conditions/regulations. ** Ultrasound training can be regional, optional or mandatory. *** Ultrasound training will be mandatory from 2025.					

A total of 232 different diagnostic ultrasound examinations and 8 ultrasound-guided procedures were identified in the brainstorming phase and included for assessment in the 1 ^st^ Delphi round. The Delphi panel reduced the list to 167 diagnostic ultrasound examinations and 7 ultrasound-guided procedures that required either a basic or advanced level of scanning competence ( **Supplemental Table 1** provides the results from the 1 ^st^ Delphi round). In the 2 ^nd^ Delphi round, the list was further reduced to 73 diagnostic examinations and 3 procedures that 65% of panel members included in a basic core curriculum ( **Supplemental Table 2** ). The 3 ^rd^ Delphi round resulted in a final list of 40 diagnostic ultrasound examinations that 75% of panel members included in a basic core curriculum ( Table 3 ; **Supplemental Tables 3 and 4** ). Seventeen diagnostic ultrasound examinations reached more than a 90% agreement: Abdominal aortic aneurysm, intraperitoneal fluid in Morison’s pouch, intraperitoneal fluid in the subphrenic space, gallbladder stone, acute cholecystitis, congested gallbladder, hydronephrosis, presence of urine in the urinary bladder, residual urine, pleural effusion or hemothorax, knee joint effusion, subcutaneous abscess, subcutaneous foreign body, subcutaneous hematoma, intrauterine pregnancy, fluid within the cul-de-sac (pouch of Douglas), and deep vein thrombosis.

**Table:** **Table 3** Agreement in final Delphi round.

Area	Ultrasound examination	Agreement	Likert score				Kappa*
			Mean (SD)	Median	Min.	Max.	
**Abdomen**	Abdominal aortic aneurysm	100.00%	4.95 (0.21)	5	4	5	0.26
	Intraperitoneal fluid in Morison’s pouch	96.43%	4.82 (0.51)	5	2	5	0.51
	Intraperitoneal fluid in the subphrenic space	95.24%	4.75 (0.58)	5	2	5	0.57
**Gallbladder**	Gallbladder stone	100.00%	4.98 (0.15)	5	4	5	0.20
	Acute cholecystitis	100.00%	4.90 (0.29)	5	4	5	0.27
	Congested gallbladder	91.67%	4.48 (0.69)	5	2	5	0.32
	Gallbladder wall polyp	82.14%	4.24 (0.89)	4	2	5	0.52
	Dilatation of intrahepatic bile ducts	77.38%	3.95 (1.10)	4	1	5	0.75
**Urinary tract**	Hydronephrosis	98.81%	4.93 (0.30)	5	3	5	0.48
	Presence of urine in urinary bladder	100.00%	4.95 (0.21)	5	4	5	0.14
	Residual urine	97.62%	4.86 (0.47)	5	2	5	0.31
	Foley catheter in urinary bladder	88.10%	4.52 (0.80)	5	2	5	0.55
	Stones (calculi) in the urinary bladder	86.90%	4.37 (0.86)	5	2	5	0.60
	Stones (calculi) in the kidney	80.95%	4.19 (1.08)	5	1	5	0.75
	Focal simple cystic lesion in the kidney	85.71%	4.38 (0.93)	5	1	5	0.68
	Ureteral obstruction (Dilated ureter)	76.19%	4.07 (1.12)	4	1	5	0.60
**Liver**	Steatotic liver (NAFLD/MASLD)	83.33%	4.13 (1.11)	4	1	5	0.77
	Hepatomegaly	80.95%	4.08 (1.15)	4	1	5	0.76
	Focal cystic liver lesion (liver cyst)	80.95%	4.08 (1.10)	4	1	5	0.74
**Spleen**	Enlarged spleen	83.33%	4.21 (1.09)	5	1	5	0.74
**Heart**	Pericardial effusion/cardiac tamponade	89.29%	4.48 (0.81)	5	2	5	0.54
**Lungs**	Pleural effusion or hemothorax	96.43%	4.76 (0.59)	5	2	5	0.46
	Pneumothorax	88.10%	4.48 (0.80)	5	2	5	0.60
	Interstitial syndrome (e.g., pulmonary edema)	86.90%	4.33 (0.91)	5	1	5	0.64
	Pneumonia	80.95%	4.13 (0.99)	4	1	5	0.69
**MSK**	Knee joint effusion	92.86%	4.54 (0.70)	5	2	5	0.44
	Baker’s cyst	88.10%	4.44 (0.73)	5	2	5	0.50
	Achilles tendon rupture	78.57%	4.12 (0.95)	4	1	5	0.61
**Head and neck**	Pathological lymph nodes	78.57%	4.04 (1.18)	4	1	5	0.78
**Skin**	Abscess	98.81%	4.82 (0.47)	5	2	5	0.52
	Foreign body	97.62%	4.74 (0.49)	5	3	5	0.42
	Hematoma	95.24%	4.62 (0.66)	5	2	5	0.62
	Phlegmon/cellulitis	85.71%	4.33 (0.94)	5	1	5	0.57
	Fibro-lipoma (lipoma)	86.90%	4.33 (0.83)	5	2	5	0.50
**Female pelvis**	Intrauterine pregnancy	91.67%	4.61 (0.71)	5	2	5	0.64
	Fluid within the cul-de-sac (pouch of Douglas)	92.86%	4.67 (0.65)	5	2	5	0.50
	Position of intrauterine contraceptive device (IUCD)	82.14%	4.24 (1.05)	5	1	5	0.65
	Fetal heartbeat	76.19%	4.20 (1.16)	5	1	5	0.74
**Male pelvis**	Prostate hyperplasia	80.95%	4.15 (1.08)	4	1	5	0.79
**Other**	Deep vein thrombosis	91.67%	4.58 (0.75)	5	2	5	0.64
* Difference between ratings in round 2 and round 3 assessed using weighted Cohen’s Kappa with quadratic weighting.							

No further Delphi rounds were added to the study as kappa values were moderate (0.41–0.60) for 17 of the examinations and substantial (0.60–0.80) for 17 of the included examinations, representing good stability across rounds 2 and 3. In 6 examinations kappa values indicated no agreement (< 0.20) or only fair agreement (0.21–0.40) between rounds 2 and 3. However, there was a high (>90%) percentage of agreement regarding the inclusion of these in both rounds.

## Discussion

### Summary of main findings

Using the Delphi method and a 75% consensus level, our investigation into PoCUS examinations for inclusion in a basic curriculum for PCPs identified 40 applications. These applications fell within 13 anatomical regions and no ultrasound-guided procedures were included. High agreement was found among the participating PCPs with 17 of the 40 applications reaching more than 90% consensus and good stability was found for replies in consecutive rounds.

### Strengths and limitations

Panel members from 23 European countries completed all 3 rounds of the Delphi survey, thus making this one of the largest published Delphi studies with respect to the number of countries sampled and the number of participants. Previous Delphi studies on this topic 18 21 22 23 24 have included homogeneous groups of national experts, making consensus easier, but the results context-dependent. Instead, we aimed to strengthen our study by having a large heterogeneous panel of experts from across Europe representing different views and contexts. Still, given the recruitment strategy, our sample may not be representative.

In the absence of any known national register, asking panel members to estimate the percentage of PCPs performing ultrasound examinations in their countries was considered useful as a rough indicator. Figures offered by some members from countries such as Denmark, Switzerland, and Czechia were often similar, while estimates varied considerably in other countries including Italy, Spain, and Germany. This may be related to whether participants were based in urban or remote locations 12 or due to a response bias caused by a lack of familiarity with the national education of PCPs. Hence, these estimates may have limited generalizability but could be compared to results from future studies to inform trends.

All panelists in this study were experienced ultrasound teachers, and they were instructed to draw on this experience rather than contextual factors when evaluating ultrasound applications during the study. However, personal experience with ultrasound equipment, previous training, and experience performing different examinations may have influenced the panelists’ assessment of the complexity of different ultrasound examinations.

The brainstorming phase produced a diverse list of ultrasound applications, varying in detail, specificity, and scope, with overlaps across domains. To refine the list for the Delphi panel, the authors, in collaboration with the task force, edited and standardized the applications to enhance clarity and reduce redundancy while still safeguarding the views and opinion of each task force member ( **Supplemental file 1** ). However, some inconsistencies remained, and this may have influenced Delphi panel members’ decisions, potentially leading to the rejection of relevant basic applications. Future studies and training program development should consider this possible selection bias.

### Comparison with existing literature

**Fig. 2:**
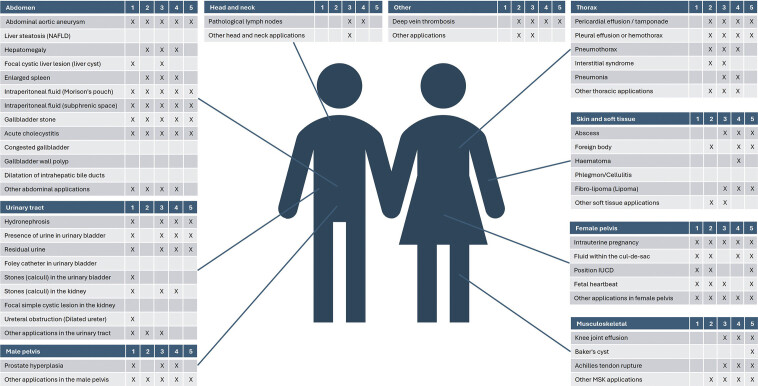
Comparing results to previous Delphi studies. Ultrasound applications included in this Delphi study compared to applications included in previous Delphi studies: 1 = Camard et al. France 2024 (reference 23), 2 = Mans et al. South Africa 2023 (reference 24), 3 = Conangla-Ferrin et al. Spain 2022 (reference 22), 4 = Homar et al. Slovenia 2020 (reference 21), and 5 = Løkkegaard et al. Scandinavian countries 2019 (reference 18).

Our findings mirror most of those identified in previous national Delphi studies 18 21 22 23 24 , even though selection criteria differed ( Fig. 2 ). This suggests that PCPs have similar perspectives on the appropriate application of PoCUS in primary care, regardless of the country of work. However, 7 of the ultrasound examinations included in our study have not been included in past Delphi studies (Foley catheter in the urinary bladder, focal simple cystic lesion in the kidney, congested gallbladder, gallbladder wall polyp, dilatation of intrahepatic bile ducts, and phlegmon/cellulitis). Depending on the study, these examinations were deselected by panelists – either because they were too difficult to perform, irrelevant in the clinical setting, or rarely encountered. Another possibility is that the examinations were not included in the list from which study panelists could choose. Our preliminary list was very exhaustive from the start, and some of the included examinations may have been redundant or overlapping and were therefore not included in other studies.

Other variations in opinion as reflected by differences between studies may be explained by differences in study methods and aims. In studies in which a selection was made based on relevance in the clinical setting, more musculoskeletal 18 , emergency 21 , and obstetric 24 applications were included in the final list. Time pressure may also influence opinions. A Scandinavian study 18 conducted prior to the COVID-19 pandemic invited office-based general practitioners to produce a list of scanning modalities to be included in a basic curriculum. Using a 67% cut-off for consensus, they identified a total of 30 applications, with 8 musculoskeletal examinations not being featured in our final list and no lung examinations making it to their final list. A French study 23 invited experienced general practitioners to screen a list of 83 PoCUS applications for relevance. They identified 17 applications with strong agreement and 19 applications with relative agreement, 14 of which are not in our final list. The South African 24 study asked PoCUS experts from family medicine departments to determine which of the 84 PoCUS examinations in the American Academy of Family Medicine (AAFM) curriculum were essential for generalists working in South African district hospitals. Using a 100% cut-off for consensus and 5 consecutive rounds, they ended up with 45 PoCUS examinations, 24 of which were not on our final list. Eleven of these were obstetric examinations. The Catalonian study 22 asked family physicians from the Catalan Society of Family and Community Medicine’s ultrasound group to identify fundamental applications and indications from a list developed from a literature review. They ended up with a list of 7 areas of application and 51 pathologies that could be assessed or discarded using PoCUS. Although definitions differed, only 17 of these overlap with our final list. Finally, Slovenian family physicians 21 were asked to specify their frequency of use of 34 listed PoCUS examinations. Twenty of these were part of our final list. Overlapping results between the Delphi studies are displayed in Fig. 2 . Hence, Delphi studies selecting examinations based on relevance in the setting, possibly mirror the patient population seen by PCPs in that specific country. Here our study represents a more generic approach and, therefore, possibly more generalizable results.

Some ultrasound examinations that may be considered clinically relevant and easy-to-perform failed to achieve sufficient consensus in the 3 ^rd^ Delphi round. This could partly be due to overlapping and competing terms in the primary selection (e.g., metastatic liver lesion versus focal solid liver lesion). If only 1 of the terms had been present, one may speculate that they could have been included. The fact that some indication areas were completely discarded during the Delphi process (e.g., thyroid, abdominal emergencies, intestine, breast, specific pediatric issues) or largely discarded (head and neck, heart, female and male pelvis) may be due to the considerable differences between the healthcare systems in the panelists’ home countries, particularly with regard to the degree of specialization of PCPs, the organization of emergency care, and the subsequent diversity of the tasks of PCPs. A third reason may be that in some countries, ultrasound examination is not the preferred standard for diagnosing, e.g., long bone fractures 25 or using rectal diameter measurement in the diagnosis of constipation in children 26 .

None of the ultrasound-guided procedures made it to the final list. However, intraarticular injection in joints, bursae injections, and ultrasound-guided venous access reached more than a 75% agreement in the 2 ^nd^ round ( **Supplemental Table 2** ), suggesting that these procedures are suitable for next-level applications.

An outlier in our study may be the inclusion of pathological neck lymph nodes (cervical lymphadenopathy) but with no other neck or thyroid examination making it into the top 40. Arguably, teaching PCPs the 7 levels required for identifying cervical lymphadenopathy is beyond inclusion in a basic curriculum and is absent in the curriculum devised by the AAFP 15 . Cervical nodes have also not been identified as a priority in other recent investigative studies 12 18 23 but are featured in the consensus recommendations of the Catalan Society of Family and Community Medicine 22 . Still, reducing this scanning modality to a PoCUS examination simply looking at enlarged cervical lymph nodes (yes/no) may be feasible and improve diagnostic accuracy in primary care compared to palpation alone.

Another outlier may be the inclusion of ‘subcutaneous hematoma’. In our study subcutaneous hematoma was included in the final list, along with abscess, foreign body, cellulitis, and lipoma, giving the PCP the ability to differentiate hematoma from other frequent conditions. However, distinguishing subcutaneous hematoma from other subcutaneous fluid collections such as seromas, abscesses, liquefied lymph nodes, and even myxoid or hemorrhagic soft-tissue sarcomas is difficult. Considering the fact that some PCPs admit to examining anatomical areas for which they have not received training 14 , high-risk areas of practice for novices must be highlighted. Training recommendations must therefore outline the scope of practice for novices in order to protect both PCPs and patients.

Our study aimed to focus solely on the complexity of performing specific PoCUS examinations. However, when creating a training curriculum for PCPs, other factors must also be taken into account: Patient consultations in primary care are short 27 , leaving little room for performing PoCUS examinations; PCPs often work in smaller office-based clinics where there are limited possibilities for direct supervision, the frequency of scans may be low 8 , and there is a low disease prevalence in the primary care patient population 28 . These factors require a curriculum comprising simple scans that are easy to learn and master, that are frequently encountered, safe to perform, relevant and cost-effective. In addition, the resolution and available software vary across ultrasound systems, as does the price of the equipment. The PoCUS curriculum for primary care physicians should align with basic device capabilities, though advancing technology and AI may expand future possibilities.

In addition, a basic training curriculum must condense redundant and overlapping items from the list into operational scanning protocols, e.g., including residual urine, the presence of urine in the bladder, and assessing prostate hypertrophy in one scanning protocol of the urinary bladder. During training, tutors may touch upon commonly encountered differential diagnoses but must be careful not to expand the curriculum too broadly or include items with a low agreement score.

The above-mentioned constraints should be taken into consideration when course providers/healthcare administrators plan how PoCUS should be implemented in national healthcare systems. For instance, PoCUS in obstetrics and gynecology is not warranted in countries where these patients are not seen by PCPs. This means that the content of an EFSUMB core curriculum can only provide a framework for the design of national curricula for PCPs. For the first time, however, a framework has been created, based on the competence level required to perform the examination by a large, balanced group of European frontline physicians experienced in ultrasound in terms of specialties and nations in an orderly consensus process.

It was anticipated that by the end of the 3 ^rd^ round, fewer than 40 ultrasound examinations would have been identified for inclusion in a basic curriculum. However, these 40 items, if all are included in a curriculum, may easily be grouped into smaller region-specific modules, which would allow PCPs to select training and assessment packages more suited to their local needs. Another way to group the items would be to develop a Basic 1 and Basic 2 curriculum, including the simple scans that more than 90% of our participants agreed upon (N=17) in the Basic 1 curriculum and then the more advanced scans that 75–90% of our participants agreed upon (N=23) in the Basic 2 curriculum. This would allow for basic core competence to be established and consolidated within the simplest and commonly encountered scans before moving on with increasing complexity.

The risks associated with PoCUS use in general practice have been linked with examinations being too advanced or too explorative 8 . This study offers guidance for the safe and reliable use of PoCUS examinations in primary care with the potential for earlier and more precise diagnosis at the frontline. However, increased use of imaging carries a risk of incidental findings, and an effort must be made to provide a pathway for how to deal with “incidental” or “unexpected findings” 29 .

## Conclusion

In our study, a total of 40 ultrasound examinations were selected collectively by an experienced panel as easy to learn and teach and, therefore, appropriate for inclusion in a basic EFSUMB PoCUS core curriculum aimed at supporting PCPs in Europe. This study provides information for the EFSUMB task force and facilitates progress toward the next stage, i.e., developing training recommendations.

## Supplementary Material

Supplementary Material Zusatzmaterial
