# Supplementary material for: A core curriculum of point-of-care ultrasound examinations for frontline physicians in primary care: results from a European Delphi study
Source: Ultraschall Med. 2025 Jun 4;47(2):160–70. doi: 10.1055/a-2590-5242 (PMC13065360; doi:10.1055/a-2590-5242)

Supplemental File 1: The brainstorming phase

A task force was appointed by EFSUMB to develop a core curriculum for European primary care doctors. During a brainstorming phase, the task force developed a comprehensive list of ultrasound scans including all scans that one or more members of the task force found appropriate in the primary care setting.

Members of the EFSUMB task force:

CJ – EFSUMB President, internist and gastroenterologist
MI – President EUVEKUS, family practitioner and pediatrician. Training curriculum in Romania
TL – Represents the Danish Society for Ultrasonography in General Practice as Vice President and a Danish University Research Group
AS – Pediatrician and member of Italian Pediatric Association Study Group organizing courses and workshops in PoCUS.
VR – Chair of the DEGUM Working Group “Ultrasound in General Practice”.
HE – Co-opted EFSUMB ExB-Member, Professional Officer of BMUS; sonographer representing the UK perspective
CC – GP in Romania
CE – EFSUMB President Elect; radiologist
KL – Family doctor and teaches ultrasonography at the Lithuanian University Health Sciences
MK – Primary physician performing sonography for referrals. Produced Polish language US website for 11,000 doctors and online courses each week (www.eduson.pol).
CAA – Associate professor, GP in training. Head of ultrasound research in Denmark

Following the brainstorming phase, content analysis was performed by the project group (TL, CAA) to allow for identification of items that were too non-specific, too similar, or could be grouped in the same category. Replies were first categorized according to the overall scanning area, e.g., musculoskeletal, abdominal, and gynecological, then according to organ, e.g., kidney, bladder, and uterus, and lastly according to condition, e.g., gall stone, living intrauterine pregnancy, or abdominal aortic aneurysm. If scanning protocols were suggested, they were subdivided into their individual constituent parts to avoid misunderstandings due to participants having different perceptions of the content of the protocol, e.g., FATE: pericardial effusion, chamber dimensions, wall thickness, or estimation of ejection fraction. Finally, the preliminary list of ultrasound examinations was sent to all task force members for comments and approval prior to the Delphi process.

Supplemental Table 1: Results of Delphi round 1

Area	Ultrasound examination	Basic	Advanced	Expert	UAD	% Basic + Advanced
Abdomen	Abdominal aortic aneurysm	76	13	5	1	93.68%
Abdomen	Intraperitoneal fluid in Morison's pouch/hepatorenal recess (ascites or bleeding)	77	11	6	1	92.63%
Abdomen	Umbilical hernia	46	40	8	1	90.53%
Abdomen	Intraperitoneal fluid in the subphrenic space or splenorenal recess (ascites or bleeding)	75	11	7	2	90.53%
Abdomen	Crural hernia/inguinal hernia	25	57	12	1	86.32%
Abdomen	Iliac artery aneurysm	33	44	12	6	81.05%
Abdomen	Incarcerated hernia	26	45	21	3	74.74%
Abdomen	Intraperitoneal free air	21	48	16	10	72.63%
Abdomen	Peritonitis	13	34	24	24	49.47%
Abdomen	Mesenteric lymphadenitis	9	36	42	8	47.37%
Abdomen	Hypertrophic pyloric stenosis (infants)	7	35	40	13	44.21%
Abdomen	Gastric emptying disorder	11	21	32	31	33.68%
Abdomen	Hiatal hernia	10	22	29	34	33.68%
Abdomen	Mesenteric artery occlusion	4	19	61	11	24.21%
Abdomen	Mesenteric vein thrombosis	3	18	61	13	22.11%
Abdomen	Gastroesophageal reflux disease (GERD)	6	9	18	62	15.79%
Abdomen	Gastric/duodenal ulcer	1	14	27	53	15.79%
Abdomen	Gastritis	5	8	20	62	13.68%
Adrenal glands	Focal cystic adrenal lesion	15	28	36	16	45.26%
Adrenal glands	Focal solid adrenal lesion (tumor)	12	28	39	16	42.11%

Adrenal glands	Adrenal hyperplasia	10	25	41	19	36.84%
Adrenal glands	Adrenal hemorrhage	7	18	48	22	26.32%
Gallbladder	Acute cholecystitis	67	24	4	0	95.79%
Gallbladder	Gallbladder stone	88	3	4	0	95.79%
Gallbladder	Congested gallbladder	62	27	5	1	93.68%
Gallbladder	Gallbladder wall polyp	46	42	6	1	92.63%
Gallbladder	Focal gallbladder wall thickening	44	40	9	2	88.42%
Gallbladder	Dilatation of intrahepatic bile ducts	36	45	13	1	85.26%
Gallbladder	Common bile duct disease	18	60	16	1	82.11%
Gallbladder	Extrahepatic bile duct dilatation	28	44	20	3	75.79%
Intestines	Appendicitis	11	60	21	3	74.74%
Intestines	Diverticulitis	13	48	24	10	64.21%
Intestines	Ileus	21	39	22	13	63.16%
Intestines	Segmental or general intestinal wall thickening	7	33	41	14	42.11%
Intestines	Small intestine disease with hyperperistalsis	11	22	41	21	34.74%
Intestines	Localized intestinal wall thickening	7	25	49	14	33.68%
Intestines	Perforated cecum	4	28	43	20	33.68%
Intestines	Perforated colon	4	27	44	20	32.63%
Rectum	Increased rectal diameter (constipation in children)	26	26	15	27	55.32%
Rectum	Anal/rectal abscess	16	30	26	22	48.94%
Rectum	Anal fissure	6	16	16	56	23.40%
Rectum	Hemorrhoidal disease	11	11	14	58	23.40%
Urinary tract	Presence of urine in urinary bladder	88	3	3	0	96.81%
Urinary tract	Hydronephrosis	80	10	3	1	95.74%
Urinary tract	Residual urine	85	5	4	0	95.74%
Urinary tract	Focal simple cystic lesion in the kidney	71	18	4	1	94.68%
Urinary tract	Stones (calculi) in the urinary bladder	68	21	4	1	94,68%

Urinary tract	Foley catheter in the urinary bladder	73	14	5	2	92.55%
Urinary tract	Stones (calculi) in the kidney	47	36	9	2	88.30%
Urinary tract	Focal solid lesion in the kidney	30	48	13	3	82.98%
Urinary tract	Focal wall thickening in the urinary bladder	38	40	12	4	82.98%
Urinary tract	Diverticula In the urinary bladder	45	33	13	3	82.98%
Urinary tract	Focal complex cystic lesion in the kidney	29	48	16	1	81.91%
Urinary tract	Ureteral obstruction (dilated ureter)	39	36	16	3	79.79%
Urinary tract	Stones (calculi) in the ureter	21	50	18	5	75.53%
Urinary tract	Kidney rupture	14	47	22	11	64.89%
Urinary tract	Pyelonephritis	15	42	22	15	60.64%
Urinary tract	Urinoma	16	39	20	19	58.51%
Urinary tract	Hemorrhagic cystitis	17	28	11	38	47.87%
Urinary tract	Infection in the urinary bladder	14	23	10	47	39.36%
Liver	Hepatomegaly	58	28	6	2	91.49%
Liver	Focal cystic liver lesion (liver cyst)	64	19	10	1	88.30%
Liver	Steatotic liver (NAFLD)	50	30	12	2	85.11%
Liver	Cirrhotic liver	35	41	16	2	80.85%
Liver	Focal solid liver lesion	34	42	16	2	80.85%
Liver	Metastatic liver	39	33	20	2	76.60%
Liver	Hepatic venous congestion	23	44	22	5	71.28%
Liver	Liver rupture/traumatic liver injury	20	44	23	7	68.09%
Liver	Hepatitis	7	38	18	31	47.87%
Pancreas	Dilated main pancreatic duct	18	45	27	4	67.02%
Pancreas	Focal cystic pancreatic lesion	20	38	32	4	61.70%
Pancreas	Peripancreatic fluid	16	39	34	5	58.51%
Pancreas	Focal solid pancreatic lesion	8	45	36	5	56.38%
Pancreas	Acute pancreatitis	11	39	31	13	53.19%
Pancreas	Chronic pancreatitis	7	39	38	10	48.94%
Spleen	Enlarged spleen	65	20	6	3	90.43%
Spleen	Ruptured spleen/traumatic splenic injury	30	46	13	5	80.85%
Spleen	Focal cystic splenic lesion	45	28	17	4	77.66%

Spleen	Focal solid splenic lesion	28	39	22	5	71.28%
Spleen	Diffuse splenic diseases	11	46	24	13	60.64%
Breast	Abscess in the breast	43	36	10	5	84.04%
Breast	Focal cystic breast lesion (cyst)	41	36	14	3	81.91%
Breast	Mastitis	19	40	18	17	62.77%
Breast	Breast wound	19	29	12	34	51.06%
Breast	Gynecomastia	25	19	26	24	46.81%
Breast	Fibrocystic breast disease	7	32	38	17	41.49%
Breast	Fibroadenoma	10	28	38	18	40.43%
Breast	Adenoma	5	28	41	20	35.11%
Breast	Breast metastases	4	28	39	23	34.04%
Breast	Mammary duct ectasia	10	22	38	24	34.04%
Breast	Nipple adenoma	7	18	46	23	26.60%
Breast	Epithelial hyperplasia (ductal and lobular lesions)	2	22	42	28	25.53%
Breast	Ductal carcinoma	4	19	47	24	24.47%
Breast	Phyllodes tumor	2	20	41	31	23.40%
Breast	Lobular carcinoma	6	15	51	22	22.34%
Breast	Lymphoma	3	17	49	25	21.28%
Breast	Hamartoma	3	17	48	26	21.28%
Breast	Stromal breast cancer	5	14	50	25	20.21%
Breast	Granular cell tumor	3	14	46	31	18.09%
Heart	Pericardial effusion/cardiac tamponade	58	21	9	5	84.95%
Heart	Increased dimensions of left cardiac chambers	22	52	14	5	79.57%
Heart	Cardiac arrest	50	21	6	16	76.34%
Heart	Increased dimensions of right cardiac chambers	23	47	18	5	75.27%
Heart	General contractility disorder, e.g. hypokinesia	14	48	25	6	66.67%
Heart	Arrhythmia	29	30	12	22	63.44%
Heart	Fluid status/hypovolemia	26	32	26	9	62.37%

Heart	Decreased ventricular contractility (left or right)	15	40	31	7	59.14%
Heart	Focal/regional contractility disorder, e.g. hypokinesia	4	48	35	6	55.91%
Heart	Valvular regurgitation	8	39	41	5	50.54%
Heart	Valvular stenosis	8	36	44	5	47.31%
Heart	Paradoxical septal motion	7	33	47	6	43.01%
Heart	Intracardiac thrombi	14	26	46	7	43.01%
Heart	Left ventricular outflow obstruction	4	33	49	7	39.78%
Heart	Diastolic dysfunction	2	34	50	7	38.71%
Heart	Atrial and ventricular septal defect	4	28	55	6	34.41%
Lungs	Pleural effusion or hemothorax	73	14	3	3	93.55%
Lungs	Pneumothorax	51	33	5	4	90.32%
Lungs	Interstitial syndrome (e.g., pulmonary edema)	47	35	7	4	88.17%
Lungs	Pneumonia	44	36	9	4	86.02%
Lungs	Subpleural pulmonary consolidation	42	35	11	5	82.80%
Lungs	Lung consolidation	44	30	13	6	79.57%
MSK	Knee joint effusion	68	19	3	3	93.55%
MSK	Baker's cyst	60	27	5	1	93.55%
MSK	Achilles tendon rupture	49	32	9	3	87.10%
MSK	Knee hematoma	61	19	8	5	86.02%
MSK	Olecranon bursitis	55	24	9	5	84.95%
MSK	Ankle joint effusion	40	37	11	5	82.80%
MSK	Subacromial bursitis	31	45	9	8	81.72%
MSK	Bone fracture in the patella	37	38	8	10	80.65%
MSK	Trochanteric bursitis	24	50	10	9	79.57%
MSK	Popliteal aneurysm	27	47	13	6	79.57%
MSK	Achilles tendinopathy	40	34	14	5	79.57%
MSK	Long tubular bone fractures (tibia, fibula, femur)	40	34	6	13	79.57%
MSK	Elbow joint effusion	35	39	12	7	79.57%

MSK	Clavicular fracture	41	33	6	13	79.57%
MSK	Hip joint effusion	31	42	14	6	78.49%
MSK	Biceps tendon synovitis	38	35	12	8	78.49%
MSK	Biceps tendon rupture	31	42	14	6	78.49%
MSK	Glenohumeral joint effusion	34	38	13	8	77.42%
MSK	Wrist joint effusion	34	38	12	9	77.42%
MSK	Rib fracture	35	36	11	11	76.34%
MSK	Acromio-clavicular joint effusion	36	33	14	10	74.19%
MSK	Long tubular bone fractures (radius, ulna, humerus)	35	34	9	15	74.19%
MSK	Small joint effusion of the hand	21	47	18	7	73.12%
MSK	Quadriceps tendinopathy	16	51	17	9	72.04%
MSK	Small joint effusion of the foot	24	43	20	6	72.04%
MSK	Patellar tendinopathy	21	45	18	9	70.97%
MSK	Carpal tunnel syndrome (median nerve)	16	49	16	12	69.89%
MSK	Sternum fracture	25	40	14	14	69.89%
MSK	Lateral or medial epicondylitis (elbow)	22	42	15	14	68.82%
MSK	Dislocation of the acromioclavicular/ sternoclavicular joints	17	46	19	11	67.74%
MSK	Shoulder rotator cuff tear	18	45	24	6	67.74%
MSK	Fascia plantaris tendinopathy	21	40	20	12	65.59%
MSK	Gout arthritis	14	46	20	13	64.52%
MSK	Shoulder rotator cuff tendinopathy	14	46	24	9	64.52%
MSK	Abscess in the psoas muscle	12	45	31	5	61.29%
MSK	Fractures of the foot skeleton	14	42	22	15	60.22%
MSK	Sever’s disease (calcaneal apophysitis)	12	39	23	19	54.84%
MSK	Fractures of the hand skeleton	14	37	25	17	54.84%
MSK	Osgood-Schlatter disease (Apophysitis tuberositas tibiae)	17	31	26	19	51.61%
MSK	Skull fracture (head)	11	31	16	35	45.16%
MSK	Congenital hip dysplasia (infants)	8	30	45	10	40.86%

Head and neck	Pathological lymph nodes	25	46	16	6	76.34%
Head and neck	Cysts (dermoid, teratoma)	19	41	21	12	64.52%
Head and neck	Sialolithiasis	17	38	27	11	59.14%
Head and neck	Sialadenitis	15	37	27	14	55.91%
Head and neck	Cystic hygroma	15	35	21	22	53.76%
Head and neck	Thyroglossal duct cyst	11	33	32	17	47.31%
Head and neck	Focal cystic parathyroid lesion	13	23	43	14	38.71%
Head and neck	Focal solid parathyroid lesion	12	23	45	13	37.63%
Head and neck	Laryngocele	1	28	32	32	31.18%
Thyroid	Focal cystic lesion (thyroid cyst)	55	25	11	2	86.02%
Thyroid	Goiter	45	25	17	6	75.27%
Thyroid	Focal suspicious nodule	33	36	20	4	74.19%
Thyroid	Macronodular goiter	35	33	17	8	73.12%
Thyroid	Thyroiditis	31	36	20	6	72.04%
Thyroid	Colloid nodule	31	33	23	6	68.82%
Thyroid	Thyroid malformations	19	36	32	6	59.14%
Thyroid	Hyperplastic nodule	12	40	29	12	55.91%
Thyroid	Follicular adenomas	13	38	31	11	54.84%
Thyroid	Subacute thyroiditis nodule	8	41	31	13	52.69%
Thyroid	Anaplastic thyroid cancer	5	33	34	21	40.86%
Thyroid	Follicular carcinoma	4	30	37	22	36.56%
Thyroid	Papillary carcinoma	3	31	34	25	36.56%
Thyroid	Thyroid metastasis	2	32	40	19	36.56%
Thyroid	Medullary carcinoma	2	28	36	27	32.26%
Thyroid	Thyroid lymphoma	1	29	40	23	32.26%

Thyroid	Hurthle cell carcinoma	1	25	37	30	27.96%
Skin	Hematoma	73	18	2	0	97.85%
Skin	Foreign body	58	33	2	0	97.85%
Skin	Abscess	76	14	3	0	96.77%
Skin	Phlegmon/cellulitis	48	41	1	3	95.70%
Skin	Fibrolipoma (lipoma)	54	32	5	2	92.47%
Skin	Epidermal inclusion cyst	41	39	10	3	86.02%
Skin	Prominent xiphoid process	31	36	6	20	72.04%
Female pelvis	Intrauterine pregnancy	70	15	3	5	91.40%
Female pelvis	Fluid within the cul-de-sac (pouch of Douglas)	62	20	6	5	88.17%
Female pelvis	Position of intrauterine contraceptive device (IUCD)	63	19	6	5	88.17%
Female pelvis	Number of embryos	45	30	11	7	80.65%
Female pelvis	Thickened endometrium	39	35	12	7	79.57%
Female pelvis	Fetal heartbeat	55	19	12	7	79.57%
Female pelvis	Ovarian cystic lesion (ovarian cyst)	37	36	15	5	78.49%
Female pelvis	Fetal position	46	27	14	6	78.49%
Female pelvis	Focal solid lesion in the uterus (Uterine mass)	26	46	15	6	77.42%
Female pelvis	Gestational age (CRL first trimester)	36	31	16	10	72.04%
Female pelvis	Absent or underdeveloped uterus	27	34	23	9	65.59%
Female pelvis	Ovarian solid lesion/adnexal mass	18	41	26	8	63..44%
Female pelvis	Ruptured ovarian cyst	13	46	26	8	63.44%
Female pelvis	Ectopic pregnancy	12	45	26	10	61.29%
Female pelvis	Tubo-ovarian abscess	11	38	33	11	52.69%
Female pelvis	Hematocolpos	10	37	25	21	50.54%
Female pelvis	Salpingitis	6	35	37	15	44.09%
Female pelvis	Ovarian torsion	7	28	47	11	37.63%
Female pelvis	Round ligament varices	5	27	36	25	34.41%
Male pelvis	Inguinal/scrotal hernia	29	54	8	2	89.25%
Male pelvis	Hydrocele	44	39	6	4	89.25%

Male pelvis	Prostate hyperplasia	53	26	10	4	84.95%
Male pelvis	Varicocele	28	51	11	3	84.95%
Male pelvis	Focal scrotal/testicular cystic lesion	34	38	16	5	77.42%
Male pelvis	Epididymal simple cyst	30	40	18	5	75.27%
Male pelvis	Focal scrotal/testicular solid lesion	27	42	19	5	74.19%
Male pelvis	Epididymitis	16	48	15	14	68.82%
Male pelvis	Focal cystic lesion in the prostate	28	34	24	7	66.67%
Male pelvis	Scrotal bleeding	20	41	21	11	65.59%
Male pelvis	Focal solid lesion in the prostate	14	46	26	7	64.52%
Male pelvis	Orchitis	12	43	21	17	59.14%
Male pelvis	Testicular torsion	13	40	33	7	56.99%
Male pelvis	Prostatitis	11	36	23	23	50.54%
Other	Deep vein thrombosis	51	38	4	0	95.70%
Other	Carotid artery plaques	38	38	16	1	81.72%
Other	Increased cranial pressure (optic nerve)	14	29	33	17	46.24%
Other	Hydrocephalus (children)	8	18	42	25	27.96%
Procedure	Intra-articular injection (joints)	27	44	16	6	76.34%
Procedure	Bursae injections	23	47	18	5	75.27%
Procedure	Ultrasound-guided arterial/venous access	34	36	20	3	75.27%
Procedure	Near-tendon injections	18	50	21	4	73.12%
Procedure	Diagnostic puncture of joints	19	48	19	7	72.04%
Procedure	Paracentesis (abdomen)	23	38	26	6	65.59%
Procedure	Thoracocentesis (lung)	21	37	27	8	62.37%
Procedure	Diagnostic puncture of breast	5	37	41	10	45.16%

Supplemental Table 2: Results of Delphi round 2

Area	Ultrasound examination	(1) Strongly agree	(2) Agree	(3) Neither agree nor disagree	(4) Disagree	(5) Strongly disagree	% Strongly agree + agree
Abdomen	Abdominal aortic aneurysm	79	9	0	0	0	100.00%
	Intraperitoneal fluid in Morison's pouch	75	10	1	2	0	96.59%
	Intraperitoneal fluid in the subphrenic space	71	13	2	2	0	95.45%
	Iliac artery aneurysm	29	34	14	11	0	71.59%
	Umbilical hernia	28	29	21	9	1	64.77%
	Incarcerated hernia	31	25	17	9	6	63.64%
	Crural hernia/inguinal hernia	28	25	21	12	2	60.23%
	Intraperitoneal free air	25	24	20	11	8	55.68%
Gallbladder	Gallbladder stone	84	3	1	0	0	98.86%
	Acute cholecystitis	77	9	1	0	1	97.73%
	Congested gallbladder	51	31	5	0	1	93.18%
	Gallbladder wall polyp	39	32	9	6	2	80.68%
	Focal gallbladder wall thickening	45	19	13	9	2	72.73%
	Dilatation of intrahepatic bile ducts	40	23	9	12	4	71.59%
	Common bile duct disease	31	28	14	10	5	67.05%
	Extrahepatic bile duct dilatation	34	25	15	9	5	67.05%
Intestines	Appendicitis	36	22	11	11	8	65.91%
	Diverticulitis	29	22	15	17	5	57.95%
	Ileus	33	17	21	15	2	56.82%
	Increased rectal diameter (child constipation)	22	23	24	15	4	51.14%

Urinary tract	Hydronephrosis	77	10	0	1	0	98.86%
	Presence of urine in urinary bladder	81	5	1	1	0	97.73%
	Residual urine	71	14	3	0	0	96.59%
	Foley catheter in urinary bladder	51	27	6	3	1	88.64%
	Stones (calculi) in the urinary bladder	46	31	9	2	0	87.50%
	Stones (calculi) in the kidney	54	20	8	4	2	84.09%
	Focal simple cystic lesion in the kidney	53	21	9	3	2	84.09%
	Ureteral obstruction (dilated ureter)	36	31	14	5	2	76.14%
	Focal solid lesion in the kidney	34	29	11	7	7	71.59%
	Diverticula in the urinary bladder	29	32	18	6	3	69.32%
	Stones (calculi) in the ureter	25	35	12	10	6	68.18%
	Focal wall thickening in the urinary bladder	31	27	19	6	5	65.91%
	Focal complex cystic lesion in the kidney	33	24	13	10	8	64.77%
	Pyelonephritis	26	29	19	8	6	62.50%
	Kidney rupture	18	29	16	18	7	53.41%
	Urinoma	18	23	23	17	7	46.59%
Liver	Steatotic liver (NAFLD)	42	27	9	7	3	78.41%
	Hepatomegaly	42	25	8	11	2	76.14%
	Focal cystic liver lesion (liver cyst)	40	27	12	7	2	76.14%
	Metastatic liver	34	30	7	11	6	72.73%
	Cirrhotic liver	34	28	10	13	3	70.45%
	Focal solid liver lesion	34	27	11	7	9	69.32%
	Hepatic venous congestion	21	32	15	16	4	60.23%

	Liver rupture/traumatic liver injury	27	23	14	16	8	56.82%
Pancreas	Dilated main pancreatic duct	29	25	12	11	11	61.36%
	Acute pancreatitis	23	25	13	17	10	54.55%
	Focal solid pancreatic lesion	23	24	10	16	15	53.41%
	Peripancreatic fluid	19	27	13	20	9	52.27%
	Focal cystic pancreatic lesion	23	22	10	21	12	51.14%
Spleen	Enlarged spleen	51	19	8	8	2	79.55%
	Ruptured spleen/traumatic splenic injury	40	15	12	16	5	62.50%
	Focal cystic splenic lesion	23	22	17	17	9	51.14%
	Focal solid splenic lesion	21	24	15	16	12	51.14%
	Diffuse splenic diseases	18	19	21	22	8	42.05%
Breast	Abscess in the breast	34	35	10	7	2	78.41%
	Mastitis	18	33	17	13	7	57.95%
	Focal cystic breast lesion (cyst)	25	22	17	18	6	53.41%
	Breast wound	11	20	25	22	10	35.23%
Heart	Pericardial effusion/cardiac tamponade	49	31	5	2	1	90.91%
	Increased dimensions of left cardiac chambers	17	38	13	16	4	62.50%
	Increased dimensions of right cardiac chambers	22	30	16	16	4	59.09%
	Fluid status/hypovolemia	29	23	16	15	5	59.09%
	Cardiac arrest	29	20	14	13	12	55.68%
	Decreased ventricular contractility (left or right)	12	30	18	19	9	47.73%
	General contractility disorder, e.g., hypokinesia	13	26	18	22	9	44.32%
	Arrhythmia	15	20	22	17	14	39.77%

	Focal/regional contractility disorder, e.g., hypokinesia	7	23	23	25	10	34.09%
	Valvular regurgitation	6	23	19	25	15	32.95%
Lungs	Pleural effusion or hemothorax	61	26	0	0	1	98.86%
	Pneumothorax	52	29	4	1	2	92.05%
	Interstitial syndrome (e.g., pulmonary edema)	44	30	11	2	1	84.09%
	Pneumonia	42	32	9	5	0	84.09%
	Lung consolidation	36	29	15	6	2	73.86%
	Subpleural pulmonary consolidation	36	27	17	6	2	71.59%
MSK	Knee joint effusion	40	34	8	5	0	85.06%
	Baker's cyst	41	32	8	6	0	83.91%
	Achilles tendon rupture	35	35	9	8	0	80.46%
	Knee hematoma	31	33	14	8	1	73.56%
	Achilles tendinopathy	22	38	18	8	1	68.97%
	Long tubular bone fractures (tibia, fibula, femur)	18	39	12	14	4	65.52%
	Trochanteric bursitis	17	38	16	14	2	63.22%
	Ankle joint effusion	27	28	19	12	1	63.22%
	Hip joint effusion	20	34	21	11	1	62.07%
	Bone fracture in the patella	17	37	16	13	4	62.07%
	Popliteal aneurysm	24	29	19	14	1	60.92%
	Gout arthritis	13	29	22	19	4	48.28%
	Patellar tendinopathy	18	22	25	17	5	45.98%
	Quadriceps tendinopathy	16	22	23	20	6	43.68%
	Fractures of the foot skeleton	11	27	21	17	11	43.68%
	Osgood-Schlatter disease (Apophysitis tuberositas tibiae)	16	21	23	20	7	42.53%
	Fascia plantaris tendinopathy	15	20	30	14	8	40.23%

	Small joint effusion of the foot	14	21	27	20	5	40.23%
	Abscess in the psoas muscle	9	24	24	21	9	37.93%
	Sever's disease (calcaneal apophysitis)	11	15	34	18	9	29.89%
	Rib fracture	27	37	12	10	1	73.56%
	Clavicular fracture	23	38	13	11	2	70.11%
	Biceps tendon rupture	26	33	19	7	2	67.82%
	Olecranon bursitis	27	32	15	11	2	67.82%
	Glenohumeral joint effusion	26	32	15	13	1	66.67%
	Subacromial bursitis	29	29	16	11	2	66.67%
	Long tubular bone fractures (radius, ulna, humerus)	20	36	15	14	2	64.37%
	Acromio-clavicular joint effusion	25	27	18	15	2	59.77%
	Elbow joint effusion	24	28	21	12	2	59.77%
	Wrist joint effusion	20	31	19	13	4	58.62%
	Shoulder rotator cuff tear	25	25	21	12	4	57.47%
	Biceps tendon synovitis	26	23	22	13	3	56.32%
	Shoulder rotator cuff tendinopathy	26	22	19	15	5	55.17%
	Lateral or medial epicondylitis (elbow)	18	26	21	17	5	50.57%
	Dislocation of the acromioclavicular/sternoclavicular joints	10	33	20	22	2	49.43%
	Fractures of the hand skeleton	15	28	22	18	4	49.43%
	Carpal tunnel syndrome (median nerve)	18	24	20	16	9	48.28%
	Sternum fracture	13	28	24	18	4	47.13%
	Small joint effusion of the hand	14	26	20	23	4	45.98%

Head and neck	Pathological lymph nodes	38	30	4	9	6	78.16%
	Sialadenitis	16	29	20	15	7	51.72%
	Sialolithiasis	18	27	20	14	8	51.72%
	Cysts (dermoid, teratoma)	22	19	25	13	8	47.13%
	Cystic hygroma	15	16	32	18	6	35.63%
Thyroid	Focal cystic lesion (thyroid cyst)	33	29	14	5	6	71.26%
	Thyroiditis	29	32	10	9	7	70.11%
	Focal suspicious nodule	31	28	13	7	8	67.82%
	Goiter	33	25	13	12	4	66.67%
	Macronodular goiter	26	22	16	16	7	55,17%
	Colloid nodule	24	24	15	16	8	55.17%
	Hyperplastic nodule	15	23	22	17	10	43.68%
	Subacute thyroiditis nodule	15	23	22	16	11	43.68%
	Follicular adenomas	17	20	22	18	10	42.53%
	Thyroid malformations	15	19	22	20	11	39.08%
Skin	Abscess	65	21	1	0	0	98.85%
	Foreign body	53	31	2	1	0	96.55%
	Hematoma	56	27	3	1	0	95.40%
	Phlegmon/cellulitis	39	37	8	3	0	87.36%
	Fibrolipoma (lipoma)	41	33	9	3	1	85.06%
	Epidermal inclusion cyst	33	20	26	5	3	60.92%
	Prominent xiphoid process	18	17	29	15	8	40.23%
Female pelvis	Intrauterine pregnancy	54	22	5	4	1	88.37%
	Fluid within the cul-de-sac (pouch of Douglas)	60	15	3	7	1	87.21%
	Position of intrauterine contraceptive device (IUCD)	42	23	10	11	0	75.58%
	Fetal heartbeat	45	17	10	9	5	72.09%
	Number of embryos	34	23	10	15	4	66.28%
	Ovarian cystic lesion (ovarian cyst)	30	23	18	11	4	61.63%

	Ectopic pregnancy	31	21	13	15	6	60.47%
	Thickened endometrium	26	23	22	12	3	56.98%
	Focal solid lesion in the uterus (uterine mass)	20	26	21	12	7	53.49%
	Gestational age (CRL first trimester)	30	14	20	16	6	51.16%
	Fetal position	27	17	20	15	7	51.16%
	Ruptured ovarian cyst	21	21	24	12	8	48.84%
	Ovarian solid lesion/adnexal mass	18	23	19	17	9	47.67%
	Tubo-ovarian abscess	12	23	25	17	9	40.70%
	Absent or underdeveloped uterus	14	19	23	19	11	38.37%
	Hematocolpos	10	15	32	22	7	29.07%
Male pelvis	Prostate hyperplasia	37	26	10	9	4	73.26%
	Hydrocele	25	36	13	10	2	70.93%
	Inguinal/scrotal hernia	30	27	17	12	0	66.28%
	Varicocele	18	34	19	13	2	60.47%
	Testicular torsion	22	28	16	12	8	58.14%
	Epididymitis	15	34	20	11	6	56.98%
	Focal scrotal/testicular solid lesion	13	34	16	17	6	54.65%
	Focal scrotal/testicular cystic lesion	14	31	20	15	6	52.33%
	Epididymal simple cyst	16	27	17	17	9	50.00%
	Orchitis	17	26	21	14	8	50.00%
	Scrotal bleeding	13	24	26	18	5	43.02%
	Focal cystic lesion in the prostate	16	18	23	17	12	39.53%
	Prostatitis	14	17	25	18	12	36.05%
	Focal solid lesion in the prostate	16	14	23	20	13	34.88%
Other	Deep vein thrombosis	61	15	5	3	2	88.37%

	Carotid artery plaques	36	20	13	9	8	65.12%
Procedure	Diagnostic puncture of joints	28	31	15	6	6	68.60%
	Ultrasound-guided arterial/venous access	28	31	14	7	6	68.60%
	Intra-articular injection (joints)	35	23	13	9	6	67.44%
	Bursae injections	30	21	17	13	5	59.30%
	Near-tendon injections	29	21	18	12	6	58.14%
	Paracentesis (abdomen)	21	21	15	16	13	48.84%
	Thoracocentesis (lung)	17	21	13	21	14	44.19%

Supplemental Table 3: Results of Delphi round 3

Area	Ultrasound examination	(1) Strongly agree	(2) Agree	(3) Neither agree nor disagree	(4) Disagree	(5) Strongly disagree	% Strongly agree + agree
Abdomen	Abdominal aortic aneurysm	80	4	0	0	0	100.00%
	Intraperitoneal fluid in Morison's pouch	73	8	2	1	0	96.43%
	Intraperitoneal fluid in the subphrenic space	68	12	3	1	0	95.24%
	Iliac artery aneurysm	13	32	27	12	0	53.57%
Gallbladder	Gallbladder stone	82	2	0	0	0	100.00%
	Acute cholecystitis	76	8	0	0	0	100.00%
	Congested gallbladder	48	29	6	1	0	91.67%
	Gallbladder wall polyp	40	29	10	5	0	82.14%
	Focal gallbladder wall thickening	27	35	12	8	2	73.81%
	Dilatation of intrahepatic bile ducts	29	36	10	4	5	77.38%
	Common bile duct disease	17	22	26	13	6	46.43%
	Extrahepatic bile duct dilatation	22	26	19	11	6	57.14%
Intestines	Appendicitis	30	22	15	11	6	61.90%
Urinary tract	Hydronephrosis	79	4	1	0	0	98.81%
	Presence of urine in the urinary bladder	80	4	0	0	0	100.00%
	Residual urine	75	7	1	1	0	97.62%
	Foley catheter in the urinary bladder	57	17	7	3	0	88.10%
	Stones (calculi) in the urinary bladder	47	26	6	5	0	86.90%

	Stones (calculi) in the kidney	44	24	6	8	2	80.95%
	Focal simple cystic lesion in the kidney	50	22	8	2	2	85.71%
	Ureteral obstruction (dilated ureter)	39	25	10	7	3	76.19%
	Focal solid lesion in the kidney	26	35	8	11	4	72.62%
	Diverticula in the urinary bladder	25	24	19	15	1	58.33%
	Stones (calculi) in the ureter	19	20	22	15	8	46.43%
	Focal wall thickening in the urinary bladder	21	25	16	17	5	54.76%
	Focal complex cystic lesion in the kidney	20	27	13	16	8	55.95%
Liver	Steatotic liver (NAFLD)	39	31	4	6	4	83.33%
	Hepatomegaly	39	29	4	8	4	80.95%
	Focal cystic liver lesion (liver cyst)	37	31	5	8	3	80.95%
	Metastatic liver	32	30	6	7	9	73.81%
	Cirrhotic liver	24	32	14	6	8	66.67%
	Focal solid liver lesion	25	28	12	11	8	63.10%
Spleen	Enlarged spleen	45	25	3	9	2	83.33%
Breast	Abscess in the breast	27	32	13	9	3	70.24%
Heart	Pericardial effusion/cardiac tamponade	53	22	5	4	0	89.29%
Lungs	Pleural effusion or hemothorax	69	12	1	2	0	96.43%
	Pneumothorax	53	21	7	3	0	88.10%
	Interstitial syndrome (e.g., pulmonary edema)	45	28	7	2	2	86.90%
	Pneumonia	36	32	9	5	2	80.95%
	Lung consolidation	37	22	12	11	2	70.24%

	Subpleural pulmonary consolidation	32	22	13	14	3	64.29%
MSK	Knee joint effusion	53	25	4	2	0	92.86%
	Baker's cyst	48	26	9	1	0	88.10%
	Achilles tendon rupture	35	31	12	5	1	78.57%
	Knee hematoma	29	29	18	6	2	69.05%
	Achilles tendinopathy	22	29	21	10	2	60.71%
	Long tubular bone fractures (tibia, fibula, femur)	19	28	23	10	4	55.95%
	Rib fracture	27	35	13	8	1	73.81%
	Clavicular fracture	23	32	18	10	1	65.48%
	Biceps tendon rupture	22	33	18	10	1	65.48%
	Olecranon bursitis	25	35	16	8	0	71.43%
	Glenohumeral joint effusion	20	35	17	9	3	65.48%
	Subacromial bursitis	23	28	21	9	3	60.71%
Head and neck	Pathological lymph nodes	38	28	5	9	4	78.57%
Thyroid	Focal cystic lesion (thyroid cyst)	32	29	9	8	6	72.62%
	Thyroiditis	21	33	11	13	6	64.29%
	Focal suspicious nodule	27	27	10	12	8	64.29%
	Goiter	25	28	14	11	6	63.10%
Skin	Abscess	71	12	0	1	0	98.81%
	Foreign body	64	18	2	0	0	97.62%
	Hematoma	58	22	2	2	0	95.24%
	Phlegmon/cellulitis	47	25	6	5	1	85.71%
	Fibrolipoma (lipoma)	43	30	7	4	0	86.90%
Female pelvis	Intrauterine pregnancy	60	17	5	2	0	91.67%
	Fluid within the cul-de-sac (pouch of Douglas)	63	15	5	1	0	92.86%
	Position of intrauterine contraceptive device	45	24	8	4	3	82.14%

	Fetal heartbeat	50	14	10	7	3	76.19%
	Number of embryos	29	19	21	9	6	57.14%
Male pelvis	Prostate hyperplasia	41	27	7	6	3	80.95%
	Hydrocele	29	31	12	10	2	71.43%
	Inguinal/scrotal hernia	21	27	21	12	3	57.14%
Other	Deep vein thrombosis	59	18	4	3	0	91.67%
	Carotid artery plaques	25	24	10	19	6	58.33%
Procedure	Diagnostic puncture of joints	24	31	17	10	2	65.48%
	Ultrasound-guided arterial/venous access	25	21	21	14	3	54.76%
	Intra-articular injection (joints)	21	31	15	14	3	61.90%

Supplemental Table 4

Reduction of ultrasound examinations within different application fields during the Delphi process

Application field	Number of ultrasound applications			
	Brainstorming	1st Delphi round	2nd Delphi round	3rd Delphi round
Abdomen	18	8	4	3
Adrenals	4	0	0	0
Biliary system	8	8	8	5
Intestine & rectum	12	4	1	0
Urinary tract	18	16	13	8
Liver	9	8	6	3
Pancreas	6	5	0	0
Spleen	5	5	1	1
Breast	19	4	1	0
Heart	16	10	1	1
Lung	6	6	6	4
MSK	41	39	12	3
Head & neck	9	5	1	1
Thyroid	17	10	4	0
Skin	7	7	5	5
Female pelvis	19	16	5	4
Male pelvis	14	14	3	1
Other	4	2	2	1
Procedure	8	7	3	0
All	240	174	76	40

Supplemental Figure 1: The Delphi process

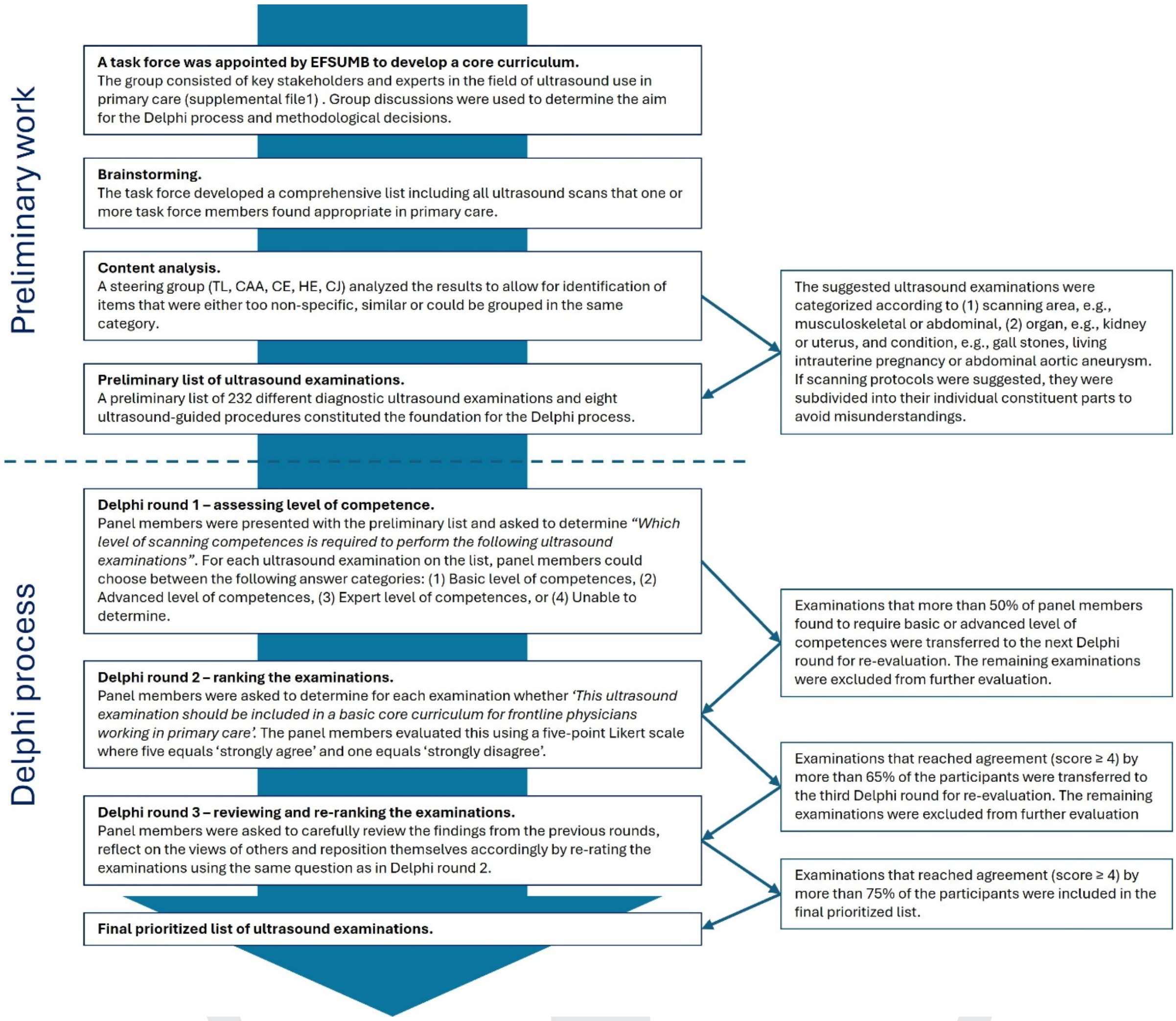

Supplement: Supplementary file 1 — Supplementary Material [file 10-1055-a-2590-5242_26044551.pdf]
